# A model and its fit lie in the eye of the beholder: Long live the sum score

**DOI:** 10.3389/fpsyg.2022.986767

**Published:** 2022-10-13

**Authors:** Peter Adriaan Edelsbrunner

**Affiliations:** Department of Humanities, Social and Political Sciences, ETH Zurich, Zurich, Switzerland

**Keywords:** latent variable, sum score, formative measurement, model equivalence, socio-constructivism

## Introduction

A common way to build scores for statistical analysis from psychological or educational scales (e.g., knowledge or intelligence tests, attitude or motivation questionnaires,) is to sum up participants' scores across all items. For example, in a questionnaire assessing knowledge about a specific topic, participants might have to answer different questions probing their content knowledge. The correct answers on all items are then summed up to build a score for analysis that is meant to represent participants' knowledge (Edelsbrunner et al., 2018). Similarly, on an intelligence test, correctly solved items might be summed to yield an overall score that is transformed into IQ estimates (Raven et al., 1962). On a questionnaire measuring need for cognition, participants might indicate their agreement with different self-descriptions on a Likert scale and their agreement is summed up (or a mean is built, which is equivalent for the arguments brought up here) across all items (Beißert et al., 2014).

Reasons to follow this practice and using sum (or mean) scores include the statistical and conceptual simplicity of building such a score: building a sum score does not require setting up an elaborate statistical model, and it might appear easily defensible to just follow this common practice without further notice.

Recent literature argues that building a sum score is, statistically and conceptually, not as innocent as it seems (Kuhfeld and Soland, 2020; McNeish and Wolf, 2020). Specifically, McNeish and Wolf (2020) argued that a sum score, although not involving any explicit statistical model in its computation, implicitly assumes a very specific and very stringent statistical model. The authors argue that whenever researchers use a sum score, they implicitly assume that a variant of factor analysis fits their data that imposes equal factor loadings and error variances across all items (the *parallel* factor model). They note that although seeming like a simple arithmetic operation, sum scoring actually *is* a simple transformation of this model. They further argue that actual factor-analytic methods have been shown to provide more accurate estimates. Consequently, they infer that using sum scores obliges researchers to engage with and test the model constraints implied by sum scores, because as in physical or social sciences, no conclusions would be endorsed without evidence (McNeish and Wolf, 2020).

I provide three counter-arguments against these views, arguing that (1) factor analysis makes meta-theoretical assumptions that are extremely stringent and seldom justified although they might not fit many constructs, (2) sum scores do not imply a factor model, because there are many other known models that imply the same data patterns as factor analysis, and (3) there is an infinite number of further models that imply the same data. Overall, this will bring me to the point that sum scores, as well as any other way to build scores, only imply the models that are theoretically defensible, not empirically.

## Meta-theoretical assumptions of factor analysis

With meta-theoretical assumptions, I describe theoretical assumptions implicit to the defining parameter structure of a class of statistical model. Factor analysis implies the meta-theoretical assumption that the latent construct that we aim at measuring is reflective (MacKenzie et al., 2005). This means that whatever the latent variable (i.e., the factor representing the latent construct; for a discussion see Maraun and Halpin, 2008) represents causally influences its indicator variables (i.e., items), and that all variance in the indicators that they do not share is measurement error. It is well-known that these assumptions do not appear appropriate for many constructs in education and psychology, theoretically (van der Maas et al., 2006) and empirically (van der Maas and Kan, 2016; van Bork et al., 2021).

Whether these assumptions are theoretically defensible can be answered by two simple questions. First, does it make sense to assume that the latent construct influences its indicators causally (e.g., if I am more motivated, this will affect me to agree more strongly with all items on a motivation-questionnaire)? If other causal pathways appear more reasonable, than the reflectivity-assumption might not be a useful representation of data. For example, instead of the construct influencing its indicators, for some constructs it might be more reasonable to assume the other causal pathway, that it, the construct being influenced by its indicators. The indicators educational background and salary raise my socio-economic status, instead of my socio-economic status raising my education and salary (Schuberth, in press). Another possibility is that there is no unifying latent construct; instead, the indicators might directly influence one another. For example, being sleepless might cause anhedonia directly, rather than both being explained by a latent construct of depression (van de Leemput et al., 2014). If any of these alternative assumptions about the relation between a construct and its indicators appears more reasonable, then factor analysis might not be a very informative model of the data-generating mechanism underlying a sum score.

A second question that might be posed to evaluate whether a factor analytic model is an appropriate representation of a construct is whether the items that were developed to capture the construct are replaceable with one another. In factor analysis, exchanging or using only a subset of indicators is supposed not to alter the meaning of the construct (White et al., 2022). If this is not the case, for example because sleeplessness and anhedonia each provide important information about depression beyond each other, than a model representing one of the outlined alternative kinds of constructs might be more appropriate than factor analysis.

## Alternative models producing the same data

The assumption that a sum score is “a simple linear transformation of a heavily constrained parallel factor model” (McNeish and Wolf, 2020) is questionable, given that a row of other statistical models have been shown to imply very similar data patterns as factor analysis. From a logical perspective, this is a converse error (“affirming the consequent”; see e.g., Martinsen, 2022): given that a constrained version of factor analysis implies a sum score, McNeish and Wolf assume that a sum score must imply factor analysis, overlooking that other models could also imply sum scores. Specifically, it can be shown mathematically that data patterns implied by factor analysis are also implied by multiple other kinds of models (e.g., Schuberth, in press). For example, a latent class analysis, modeling two classes of individuals through a categorical rather than a continuous latent variable, will generally imply the same means and variance-covariance matrix as a unidimensional factor analysis applied to the same data (Molenaar and von Eye, 1994). In addition, it has been shown that psychometric network models can produce data that are in accordance with factor analysis (van der Maas et al., 2006). Another kind of model implying equivalent data is a composite, which conceptualizes a formative rather than a reflective construct (Schuberth, in press). It stands to debate why exactly factor analysis should be applied to data to see whether it fits data and to extract factor sores, given that these alternative models, particularly if they are in better accordance with theoretical assumptions, might better capture the data-generating process.

## An infinite number of other models will fit the same data

Beyond models that are already known to mankind, we can be quite sure that many further psychometric models will be developed in the time to come. It has been shown that in principle, for any model an infinite number of alternative models exist that can fit an observed variance-covariance matrix equally well (e.g., Raykov and Marcoulides, 2001). I therefore suggest not considering any model that might be the prevalent “best practice” at one point in time as *the* data-generating model behind a sum score, or any other kinds of scores. As long as methodological research continues, it will develop many new informative models that will provide reasonable accounts of data just as well as, or even better than, factor analysis. This is especially noteworthy given that factor analysis was established more than 100 years ago (Spearman, 1904), and by now we already have a number of alternative models to choose from. Perhaps, factor analysis is commonly assumed to underlie data mostly because it has been around for so long and is a comparably well-developed approach.

## Alternative justifications of sum scores

Do I believe that these arguments free researchers from any justifications for their uses of sum scores? No. To the contrary, I would like to bring up alternative justifications. First, a sum score can be built if researchers have conceptualized the construct that they intend to represent in the score such that all indicators represent approximately equal shares of the construct. This might for example be the case if researchers aim at building an index of a construct of different skills (Van der Maas et al., 2014) or beliefs (e.g., Merk and Rosman, 2019; Schiefer et al., 2022). In this case, however, it should not be overlooked that equal weighting of indicators in an index should also be based on justification. One such justification might be that different aspects of a construct have been defined as equally important components of a theoretical model, or that they are assumed to play similarly important roles in determining an educational or psychological outcome. In such cases, a sum score provides good construct representation, that is, match between the meta-theoretical assumptions of a statistical model and the theoretical construct that it is meant to represent.

A common case probably is that researchers think about the different aspects that the construct they are intending to measure consists of, and then they develop indicators such as items in a balanced manner, such that each of these aspects is represented by the same number of items. It stands to debate why in such cases, any item(s) should be weighted more strongly than others.

Another reason to use sum scores is the aim to use scores that are as comparable across studies as possible in their constituents. If factor analysis is applied to data, factor loadings are usually estimated in a data-driven manner, such that items are put into the score with weights that have little theoretical justification. This will usually induce variation in scores that are extracted based on factor analysis across studies: if in one study, an indicator has received a strong factor weight but in another study, its weight is lower, then it is difficult to compare the meaning of these derived scores across these studies conceptually (Widaman and Revelle, in press). This might also be described as a classical issue of a bias-variance tradeoff (Yarkoni and Westfall, 2017). If scores are built in an equal manner across studies, for example by always using sum scores, then they might be biased if factor analysis might have been an unbiased model of the data-generating process. At the same time, the variance in the composition of the scores will be lower if they are always built alike. Consider however, perhaps speaking against this argument, the point that if a factor analytic model fits data well, the meaning of the latent variable does not change when indicators are replaced with one another. Consequently, also if factor loadings differ between studies, this might not change the meaning of the latent variable. This assumption might be valid as long as at least some indicators have consistent loadings across studies, implying partial measurement invariance (although measurement invariance does not imply equality of latent variables; Maraun and Heene, 2016).

Even if researchers use sum scores with theoretical but without statistical justification, this might be defensible. Educational and psychological science generally follow the maxim of an empirical science grounded in modernism (Holtz, 2020); data should inform theories. This does not, however, have to mean that all uses of data have to be empirically justified in all cases. Education and psychology are not just empirical but also socio-constructivist sciences (Guyon and Nôus, 2021). Within such a science, instead of justifying use of data exclusively empirically, researchers should be free to justify whether their use of data should be based on empirical fit, or on theoretical fit, or on both.

Finally, if researchers decide to base their use of data on empirical fit, some approaches have been developed to differentiate between data patterns that the discussed kinds of models typically produce. For example, throughout the last decades, various statistical approaches for distinguishing between factor analytic and latent class models have been introduced (De Boeck et al., 2005), as well as for distinguishing between network models and latent factors (van Bork et al., 2021).

## Take-home message

Overall, I agree with McNeish and Wolf (2020) in asking researchers for justifications for their uses of scores. I am, however, going a step back (or further, depending on the eye of the beholder) and asking researchers to first justify which of their procedures they want to justify theoretically, and which empirically, and why. Theoretical justification can be achieved through conceptualization and definition. If a construct is defined in a way such that the building of a sum score maps on this definition well (Lundberg et al., 2021), then its use is appropriate.

What will happen if in a 100 years, factor analysis is not used anymore but has been superseded by a new class of models that takes very different conceptual and statistical perspectives? And, if some variant of these new models also implies a sum score, does this then mean that sum scores will represent that new future model? Probably not. Sum scores represent only the model that has been used to create them for good theoretical reasons.

## Author contributions

The author confirms being the sole contributor of this work and has approved it for publication.

## Funding

Open access funding provided by ETH Zurich.

## Conflict of interest

The author declares that the research was conducted in the absence of any commercial or financial relationships that could be construed as a potential conflict of interest.

## Publisher's note

All claims expressed in this article are solely those of the authors and do not necessarily represent those of their affiliated organizations, or those of the publisher, the editors and the reviewers. Any product that may be evaluated in this article, or claim that may be made by its manufacturer, is not guaranteed or endorsed by the publisher.
